# The Autoinflammatory Diseases Alliance Registry of monogenic autoinflammatory diseases

**DOI:** 10.3389/fmed.2022.980679

**Published:** 2022-09-09

**Authors:** Carla Gaggiano, Antonio Vitale, Abdurrahman Tufan, Gaafar Ragab, Emma Aragona, Ewa Wiesik-Szewczyk, Djouher Ait-Idir, Giovanni Conti, Ludovica Iezzi, Maria Cristina Maggio, Marco Cattalini, Francesco La Torre, Giuseppe Lopalco, Elena Verrecchia, Amato de Paulis, Ali Sahin, Antonella Insalaco, Petros P. Sfikakis, Achille Marino, Micol Frassi, Benson Ogunjimi, Daniela Opris-Belinski, Paola Parronchi, Giacomo Emmi, Farhad Shahram, Francesco Ciccia, Matteo Piga, José Hernández-Rodríguez, Rosa Maria R. Pereira, Maria Alessio, Roberta Naddei, Alma Nunzia Olivieri, Emanuela Del Giudice, Paolo Sfriso, Piero Ruscitti, Francesca Li Gobbi, Hamit Kucuk, Jurgen Sota, Mohamed A. Hussein, Giuseppe Malizia, Karina Jahnz-Różyk, Rawda Sari-Hamidou, Mery Romeo, Francesca Ricci, Fabio Cardinale, Florenzo Iannone, Francesca Della Casa, Marco Francesco Natale, Katerina Laskari, Teresa Giani, Franco Franceschini, Vito Sabato, Derya Yildirim, Valeria Caggiano, Mohamed Tharwat Hegazy, Rosalba Di Marzo, Aleksandra Kucharczyk, Ghalia Khellaf, Maria Tarsia, Ibrahim A. Almaghlouth, Ahmed Hatem Laymouna, Violetta Mastrorilli, Laura Dotta, Luca Benacquista, Salvatore Grosso, Francesca Crisafulli, Veronica Parretti, Heitor F. Giordano, Ayman Abdel-Monem Ahmed Mahmoud, Rossana Nuzzolese, Marta De Musso, Cecilia Beatrice Chighizola, Stefano Gentileschi, Mirella Morrone, Ilenia Di Cola, Veronica Spedicato, Henrique A. Mayrink Giardini, Ibrahim Vasi, Alessandra Renieri, Alessandra Fabbiani, Maria Antonietta Mencarelli, Bruno Frediani, Alberto Balistreri, Gian Marco Tosi, Claudia Fabiani, Merav Lidar, Donato Rigante, Luca Cantarini

**Affiliations:** ^1^Department of Medical Sciences, Surgery and Neurosciences, Research Center of Systemic Autoinflammatory Diseases and Behçet's Disease Clinic, University of Siena, Siena, Italy; ^2^Division of Rheumatology, Department of Internal Medicine, Gazi University Faculty of Medicine, Ankara, Turkey; ^3^Internal Medicine Department, Rheumatology and Clinical Immunology Unit, Faculty of Medicine, Cairo University, Giza, Egypt; ^4^Faculty of Medicine, Newgiza University, 6th of October City, Egypt; ^5^Division of Gastroenterology, Ospedali Riuniti Villa Sofia-Vincenzo Cervello, Palermo, Italy; ^6^Department of Internal Medicine, Pulmonology, Allergy and Clinical Immunology, Central Clinical Hospital of the Ministry of National Defence, Military Institute of Medicine, Warsaw, Poland; ^7^Research Laboratory, Biodiversity, Biotechnology, Environment and Sustainable Development, Department of Biology, Faculty of Sciences, M'Hamed Bougara University, Boumerdes, Algeria; ^8^Pediatric Nephrology and Rheumatology Unit, Azienda Ospedaliero Universitaria (AOU) G Martino, Messina, Italy; ^9^Department of Life Sciences and Global Health, Fondazione Policlinico Universitario A. Gemelli IRCCS, Rome, Italy; ^10^University Department of Health Promotion, Mother and Child Care, Internal Medicine and Medical Specialties (PROMISE) “G. D'Alessandro”, University of Palermo, Palermo, Italy; ^11^Pediatric Clinic, University of Brescia and Spedali Civili di Brescia, Brescia, Italy; ^12^Department of Pediatrics, Pediatric Rheumatology Center, Giovanni XXIII Pediatric Hospital, University of Bari, Bari, Italy; ^13^Rheumatology Unit, Department of Emergency and Organ Transplantation, University of Bari, Bari, Italy; ^14^Rare Diseases and Periodic Fevers Research Centre, Università Cattolica del Sacro Cuore, Rome, Italy; ^15^Department of Aging, Neurological, Orthopedic and Head and Neck Sciences, Fondazione Policlinico Universitario Agostino Gemelli Istituto di Ricovero e Cura a Carattere Scientifico (IRCCS), Rome, Italy; ^16^Department of Translational Medical Sciences, Section of Clinical Immunology, University of Naples Federico II, Naples, Italy; ^17^Center for Basic and Clinical Immunology Research (CISI), World Allergy Organization (WAO) Center of Excellence, University of Naples Federico II, Naples, Italy; ^18^Division of Rheumatology, Department of Internal Medicine, Medical Faculty, Sivas Cumhuriyet University, Sivas, Turkey; ^19^Division of Rheumatology, Ospedale Pediatrico Bambino Gesù, IRCCS [European Reference Network (ERN) for Rare Immunodeficiency, Autoinflammatory and Autoimmune Diseases (RITA) Center], Rome, Italy; ^20^Joint Academic Rheumatology Program, Medical School, National and Kapodistrian University of Athens, Athens, Greece; ^21^Unit of Pediatric Rheumatology, Azienda Socio-Sanitaria Territoriale (ASST) Gaetano Pini-Centro Specialistico Ortopedico Traumatologico (CTO), Milan, Italy; ^22^Rheumatology and Clinical Immunology, Spedali Civili and Department of Clinical and Experimental Sciences, University of Brescia, Brescia, Italy; ^23^Departement of Pediatrics, Antwerp University Hospital, Edegem, Belgium; ^24^Center for Health Economics Research and Modeling Infectious Diseases, Vaccine and Infectious Diseases Institute (VAXINFECTIO), University of Antwerp, Antwerp, Belgium; ^25^Department of Rheumatology, Ziekenhuis Netwerk Antwerpen, Antwerp, Belgium; ^26^KidZ Health Castle, Universitair Ziekenhuis Brussel, Jette, Belgium; ^27^Rheumatology and Internal Medicine Department, Carol Davila University of Medicine and Pharmacy, Bucharest, Romania; ^28^Department of Experimental and Clinical Medicine, University of Florence, Florence, Italy; ^29^Behcet's Disease Unit, Rheumatology Research Center, Shariati Hospital, Tehran University of Medical Sciences, Tehran, Iran; ^30^Department of Precision Medicine, Università Degli Studi Della Campania Luigi Vanvitelli, Naples, Italy; ^31^Rheumatology Unit, Department of Medical Sciences, University and AOU of Cagliari, Cagliari, Italy; ^32^Vasculitis Research Unit, Autoinflammatory Diseases Clinical Unit, Department of Autoimmune Diseases, Hospital Clinic of Barcelona, August Pi I Sunyer Biomedical Research Institute (IDIBAPS), University of Barcelona, Barcelona, Spain; ^33^Rheumatology Division, Faculdade de Medicina, Hospital das Clínicas da Faculdade de Medicina da Universidade de São Paulo (HCFMUSP), Universidade de São Paulo, São Paulo, Brazil; ^34^Pediatric Rheumatology Unit, Department of Translational Medical Sciences, University of Naples Federico II, Naples, Italy; ^35^Department of Woman, Child and of General and Specialized Surgery, University of Campania “Luigi Vanvitelli”, Naples, Italy; ^36^Department of Maternal Infantile and Urological Sciences, Sapienza University of Rome, Rome, Italy; ^37^Rheumatology Unit, Department of Medicine (DIMED), University of Padova, Padova, Italy; ^38^Rheumatology Unit, Department of Biotechnological and Applied Clinical Sciences, University of L'Aquila, L'Aquila, Italy; ^39^Rheumatology Unit, San Giovanni di Dio Hospital, Firenze, Italy; ^40^Research Laboratory Toxicomed, Faculty of Medicine, Abou Bekr Belkaid University, Tlemcen, Algeria; ^41^Department of Immunology, Allergology, Rheumatology, Antwerp University Hospital, University of Antwerp, Antwerp, Belgium; ^42^Division of Ematology II, Ospedali Riuniti Villa Sofia-Vincenzo Cervello, Palermo, Italy; ^43^Faculté de Médecine, Service de Néphrologie, Centre Hospitalo-Universitaire Lamine Debaghine, Université Alger 1 Benyoucef Benkhedda, Alger, Algeria; ^44^Rheumatology Unit, Department of Medicine, College of Medicine, King Saud University, Riyadh, Saudi Arabia; ^45^College of Medicine Research Center, College of Medicine, King Saud University, Riyadh, Saudi Arabia; ^46^Clinical Paediatrics, Department of Molecular Medicine and Development, University of Siena, Siena, Italy; ^47^Department of Clinical Sciences and Community Health, Research Center for Adult and Pediatric Rheumatic Diseases, University of Milan, Milan, Italy; ^48^Pediatric Rheumatology Unit, Azienda Socio-Sanitaria Territoriale (ASST) Gaetano Pini Centro Specialistico Ortopedico Traumatologico (CTO), Milan, Italy; ^49^Unit of Rheumatology, Azienda Ospedaliero-Universitaria Senese, Siena, Italy; ^50^Medical Genetics, Department of Medical Biotechnologies, University of Siena, Siena, Italy; ^51^Department of Medical Biotechnologies, Med Biotech Hub and Competence Center, University of Siena, Siena, Italy; ^52^Genetica Medica, Azienda Ospedaliero-Universitaria Senese, Siena, Italy; ^53^Bioengineering and Biomedical Data Science Lab, Department of Medical Biotechnologies, University of Siena, Siena, Italy; ^54^Ophthalmology Unit, Department of Medicine, Surgery and Neurosciences, University of Siena, Siena, Italy; ^55^Familial Mediterranean Fever (FMF) Clinic, The Chaim Sheba Medical Center, Ramat-Gan, Israel; ^56^Rheumatology Unit, The Chaim Sheba Medical Center, Ramat-Gan, Israel; ^57^The Sackler Faculty of Medicine, Tel-Aviv University, Tel-Aviv, Israel

**Keywords:** autoinflammatory diseases, international registry, personalized medicine, precision medicine, rare diseases

## Abstract

**Objective:**

The present manuscript aims to describe an international, electronic-based, user-friendly and interoperable patient registry for monogenic autoinflammatory diseases (mAIDs), developed in the contest of the Autoinflammatory Diseases Alliance (AIDA) Network.

**Methods:**

This is an electronic platform, based on the Research Electronic Data Capture (REDCap) tool, used for real-world data collection of demographics, clinical, laboratory, instrumental and socioeconomic data of mAIDs patients. The instrument has flexibility, may change over time based on new scientific acquisitions, and communicate potentially with other similar registries; security, data quality and data governance are corner stones of the platform.

**Results:**

AIDA project will share knowledge and expertise on mAIDs. Since its start, 118 centers from 24 countries and 4 continents have joined the AIDA project. Fifty-nine centers have already obtained the approval from their local Ethics Committees. Currently, the platform counts 337 users (122 Principal Investigators, 210 Site Investigators, 2 Lead Investigators, and 3 data managers). The Registry collects baseline and follow-up data using 3,748 fields organized into 21 instruments, which include demographics, patient history, symptoms, trigger/risk factors, therapies, and healthcare information for mAIDs patients.

**Conclusions:**

The AIDA mAIDs Registry, acts both as a research tool for future collaborative real-life studies on mAIDs and as a service to connect all the figures called to participate. On this basis, the registry is expected to play a pivotal role in generating new scientific evidence on this group of rare diseases, substantially improving the management of patients, and optimizing the impact on the healthcare system. NCT 05200715 available at https://clinicaltrials.gov.

## Introduction

Monogenic autoinflammatory diseases (mAIDs) are a group of rare inborn errors of immunity caused by mutations in genes linked to the innate immune pathways. Constitutive overactivation of these pathways leads to increased release of monocyte- and neutrophil-derived cytokines, such as interleukin-1β, tumor necrosis factor α and type 1 interferon, which results clinically in periodic fevers and a variety of unprovoked inflammatory symptoms affecting any organ or system (1). Since the candidate gene for familial Mediterranean fever (FMF) was identified in 1997, the spectrum of mAIDs has rapidly broadened especially after the remarkable advances in molecular techniques and the extensive use of next-generation sequencing (NGS): this has led to the description of more than 50 new rare diseases in the last 10 years (2, 3). Given the heterogeneity and low prevalence of mAIDs, transnational collaboration is critical in order to collect an adequate volume of data and perform groundbreaking high-impact research. To this end, the Autoinflammatory Diseases Alliance (AIDA) Network was established in 2019 to provide an international collaborative framework for scientific research and education on rare autoinflammatory diseases (https://aidanetwork.org/en/).

In the field of rare diseases, patient registries are recognized at the international level as invaluable tools that support clinical research and orientate clinical trial design, improving both the management of patients and the overall healthcare system. According to the definition provided by the Agency for Healthcare Research and Quality, a registry is “*an organized system that uses observational study methods to collect uniform data (clinical and other) to evaluate specified outcomes for a population defined by a particular disease, condition, or exposure, and that serves one or more predetermined scientific, clinical, or policy purposes*” (4). In this respect, it is now acknowledged that a circular flow of quality actions—governance, data source identification, standardization of data, FAIRness (findable, accessible, interoperable, and reusable) of information technology (IT) infrastructures, data/information quality check, training, and audits—are seminal to create tools of significant clinical impact (5). Furthermore, clinical registries are increasingly perceived as a *service* facilitating learning networks and establishing virtuous collaborations among the scientific community, industry, regulative agencies, patients, and their support networks (6). Indeed, one of the primary aims of the European Reference Networks is “*to reinforce research and epidemiological surveillance like registries*” (7). Also, the development of the European Platform on Rare Disease Registration (EU RD), providing common services and tools to rare disease registries operating across Europe, accounts for a strategic objective of the European Commission (EC). Through the EPIRARE project (“Building Consensus and Synergies for the EU Registration of Rare Disease Patients”, www.epirare.eu) the EC seeks to harmonize registry data to ensure interoperability, standardization, and data comparability (8). In the field of rare diseases, the AIDA Network has already worked to develop different registries dedicated to specific autoinflammatory diseases and ocular inflammatory disorders (9–11).

The present manuscript aims to describe an international, electronic-based, user-friendly and interoperable patient registry for mAIDs, the Autoinflammatory Diseases Alliance Registry of Monogenic Autoinflammatory Diseases (AIDA mAIDs), developed in the context of the AIDA Network program (ClinicalTrials.gov, Identifier: NCT05200715).

## Methods

To be as comprehensive as possible, the authors adopted the registry description template questionnaire developed by Kodra et al. (12).

### Registry organization

The AIDA mAIDs registry was launched on June 25, 2020 in the context of the AIDA Network program. English is the official language of the platform.

The University of Siena is the promoter of the AIDA Project. The promoter center is responsible for the registry governance and the coordination of the AIDA Network program. The role of registry/data manager is held by the personnel of the biomedical engineering department of the University of Siena, also in charge of the IT personnel of the AIDA project. The medical staff involved in the conceptual design and development of the registry and clinical advice to enrolling centers, includes physicians with expertise in rheumatology, pediatric rheumatology, ophthalmology, immunology, gastroenterology, and medical genetics. Methodologic, statistical, ethical, legal, and social issues (ELSI) and administrative support are provided by the University of Siena.

### Type of registry

The AIDA mAIDs is an electronic-based, non-population based, physician-driven, clinical/genetic research registry (5).

### Objectives

The overarching scope of the AIDA registry for mAIDs is declined into the following general aims: (i) to share knowledge and expertise on mAIDs by linking key referral centers for these rare diseases at an international level; (ii) to raise awareness among physicians and the general population about mAIDs, improving early diagnosis; (iii) to promote future multicenter studies based on a critical volume of data from patients with mAIDs.

The AIDA mAIDs-based studies will pursue the following specific objectives:

- to depict the clinical phenotype of newly identified mAIDs and broaden the phenotypic spectrum of well-established nosological entities;
- to describe genotype-phenotype correlations;
- to describe clinical manifestations based on patients' ethnicity;
- to identify age- and gender-related factors that may affect disease onset and progression;
- to outline the long-term prognosis for these diseases and identify potential prognostic factors for negative outcomes;
- to evaluate response to different therapeutic strategies and safety of drugs;
- to recognize the possible impact of mAIDs on fertility and course of pregnancy;
- to estimate the socio-economic burden of mAIDs.

Further objectives may be added over time thanks to the flexible modular infrastructure of the registry.

### List of diseases under registration

All hereditary autoinflammatory diseases (monogenic forms) will be included in the registry. As new diseases are identified in this group, the registry shall be updated accordingly given its flexible framework. The list of genes/diseases currently included in the registry is given in Table 1. However, it is also possible to enroll patients carrying mutations in genes associated with mAIDs not yet included in the list.

**Table 1:** List of genes/diseases covered by the Autoinflammatory Diseases Alliance Registry of monogenic autoinflammatory diseases.

**Gene**	**Inheritance**	**Disease**	**ORPHA number**
ADA2 (HGNC:1839)	AR	ADA2 deficiency vasculitis	ORPHA:404553
AP1S3 (HGNC:18971)	AD	Generalized pustular psoriasis	ORPHA:247353
CARD14 (HGNC:16446)	AD	Pityriasis rubra pilaris	ORPHA:2897
IL1RN (HGNC:6000)	AR	DIRA	ORPHA:210115
IL36RN (HGNC:15561)	AR	DITRA	ORPHA:404546
LACC1 (HGNC:26789)	AR	LACC1 deficiency	–
LPIN2 (HGNC:14450)	AR	Majeed syndrome	ORPHA:77297
MEFV (HGNC:6998)	AR	FMF	ORPHA:342
	AD	PAAND	–
MVK (HGNC:7530)	AR	MKD	ORPHA:343
NLRC4 (HGNC:16412)	AD	AIFEC	ORPHA:436166
NLRP3 (HGNC:16400)	AD	CAPS-FCAS 1	ORPHA: 47045
NLRP3 (HGNC:16400)	AD	CAPS-MWS	ORPHA:575
NLRP3 (HGNC:16400)	AD	CAPS-NOMID	ORPHA:1451
NLRP12 (HGNC:22938)	AD	FCAS 2	ORPHA:247868
NOD2 (HGNC:5331)	AD	Blau syndrome	ORPHA:90340
OTULIN (HGNC:25118)	AR	ORAS	ORPHA:500062
PLCG2 (HGNC:9066)	AD	APLAID	ORPHA:324530
PLCG2 (HGNC:9066)	AD	PLAID	ORPHA:300359
POMP (HGNC:20330) PSMA3 (HGNC:9532) PSMB4 (HGNC:9541) PSMB8 (HGNC:9545) PSMB9 (HGNC:9546) PSMG2 (HGNC:24929)	AR	PRAAS	ORPHA:324977
PSTPIP1 (HGNC:9580)	AD	PAID	ORPHA:69126
RBCK1/ HOIL1 (HGNC:15864)	AR	HOIL1 deficiency	ORPHA:329173
SH3BP2 (HGNC:10825)	AD/AR	Cherubism	ORPHA:184
SLC29A3 (HGNC:23096)	AR	H syndrome	ORPHA:168569
TMEM173 (HGNC:27962)	AD	SAVI	ORPHA:425120
TNFAIP3 (HGNC:11896)	AD	Hereditary pediatric Behçet-like disease	ORPHA:476102
TNFRSF1A (HGNC:11916)	AD	TRAPS	ORPHA: 32960
Others	–	Hereditary periodic fever syndrome	ORPHA:324924

AD, autosomal dominant; ADA, adenosine deaminase; AIFEC, periodic fever infantile enterocolitis autoinflammatory syndrome; APLAID, autoinflammation, PLCG2-associated antibody deficiency and immune dysregulation; AR, autosomal recessive; CAPS, cryopyrin-associated periodic syndromes; DIRA, sterile multifocal osteomyelitis with periostitis and pustulosis; DITRA, deficiency of interleukin-36 receptor antagonist; FCAS, familial cold autoinflammatory syndrome; FMF, familial Mediterranean fever; IL, interleukin; LACC1, laccase domain containing 1; MKD, mevalonate kinase deficiency; MWS, Muckle Wells syndrome; NOMID, neonatal-onset multisystem inflammatory disease; ORAS, OTULIN-related autoinflammatory syndrome; PAAND, pyrin-associated autoinflammation with neutrophilic dermatosis; PAID, PSTPIP1-associated inflammatory diseases; PLAID, PLCG2-associated antibody deficiency and immune dysregulation; PRAAS, proteasome-associated autoinflammatory syndrome; SAVI, STING-associated vasculopathy with onset in infancy; TRAPS, tumor necrosis factor receptor-associated periodic syndrome.

### Inclusion and exclusion criteria

Patients will be enrolled on the AIDA registry if they meet all the following criteria:

Subjects diagnosed and/or treated at the AIDA network partner centers (the full list is available at https://aidanetwork.org/en/clinical-sites).
Diagnosis of mAIDs based on one of the following scenarios:
  a) the presence of a confirmatory^1^ genotype AND at least 1 among the clinical items included in the Eurofever classification criteria for cryopyrin-associated periodic syndromes (CAPS), FMF, tumor necrosis factor receptor-associated periodic syndrome (TRAPS) or mevalonate kinase deficiency (MKD) (14);
  b) the presence of a not confirmatory (see text footnote 1) genotype AND at least 2 among the clinical items included in the Eurofever classification criteria for CAPS, FMF or TRAPS;
  c) fulfillment of the Tel Hashomer or the Yalcinkaya criteria for FMF, irrespectively of the genotype (13, 15);
  d) fulfillment of the Kuemmerle-Deschner criteria for CAPS, irrespectively of the genotype (16);
  e) the presence of a clinical picture consistent with MKD or adenosine deaminase (ADA)2 deficiency AND a positive biomarker test for DADA2 (ADA2 enzyme activity) or MKD (MVK enzyme activity or mevalonic aciduria) AND an inconclusive genotype^2^ (17);
  f) the presence of a clinical picture consistent with Blau syndrome, pyogenic arthritis-pyoderma gangrenosum-acne (PAPA) syndrome, A20 haploinsufficiency, ADA2 deficiency or pyrin-associated autoinflammation with neutrophilic dermatosis (PAAND) syndrome AND the detection of a confirmatory or consistent genotype (see text footnote 2) (17);
  g) the presence of a clinical picture consistent with any monogenic autoinflammatory disease covered by the registry other than FMF, MKD, TRAPS, CAPS, Blau syndrome, PAPA syndrome, A20 haploinsufficiency, DADA2 and PAAND syndrome (Table 1) AND a confirmative genotype according to the expert clinician and genetic counseling of the reference center.

3. Willing of the subject (and/or his/her parents or legal guardian where applicable according to the national regulatory frameworks) to join the project.

Subjects carrying benign variants, likely benign variants (based on the INFEVERS classification) or no variants in genes known to be responsible for mAIDs, except for the scenarios described in 2c and 2d, are excluded from the registry (18). Specific scenarios other than those described above should be discussed with the AIDA medical staff to be considered as eligible.

### Data sources and data flow

The data sources for the registry are extracted from (i) hospital clinical charts, (ii) laboratory reports, (iii) genetic laboratory reports, (iv) instrumental exams reports, (v) patient reported outcomes. The registry system is designed to capture both retrospective and longitudinal data.

To minimize recall bias for self- or proxy-reported information in the retrospective section, participants are notified before sensitive information is asked and they are allowed to provide secondary sources of information to update or validate previously collected data. As for the prospective section of the registry, a streamlined approach through real-time ad-hoc updates to the records during routine follow-up visits is suggested. Recruiting centers are advised to enter at least one follow-up record per year or when therapeutic changes are made.

The system includes mandatory and non-mandatory fields. However, it allows editing and completing any previously unanswered fields at the user's convenience. User-friendly data collecting tools are in place, such as automatic calculation fields, calendar fields, branching logics, electronic joint count homunculus and integrated Online Mendelian Inheritance in Man (OMIM), HUGO Gene Nomenclature Committee (HGNC) and International Classification of Diseases (ICD) 10 databases. Direct explanations or Internet addresses referring to external resources useful to the interpretation of specific fields are provided when required [i.e., INFEVERS classification of gene variants (18), diagnostic/classification criteria, clinimetric scores, laboratory reference values]. Complex branching logics allow the registry system to unfold following the patient's clinical history, making data collection straightforward. Each field includes a free text area for comments and queries.

### Population under surveillance of the registry

The target population includes subjects affected by mAIDs. No specific demographic, geographic, clinical, or genetic determinants are foreseen.

### Geographic coverage

**Figure 1:**
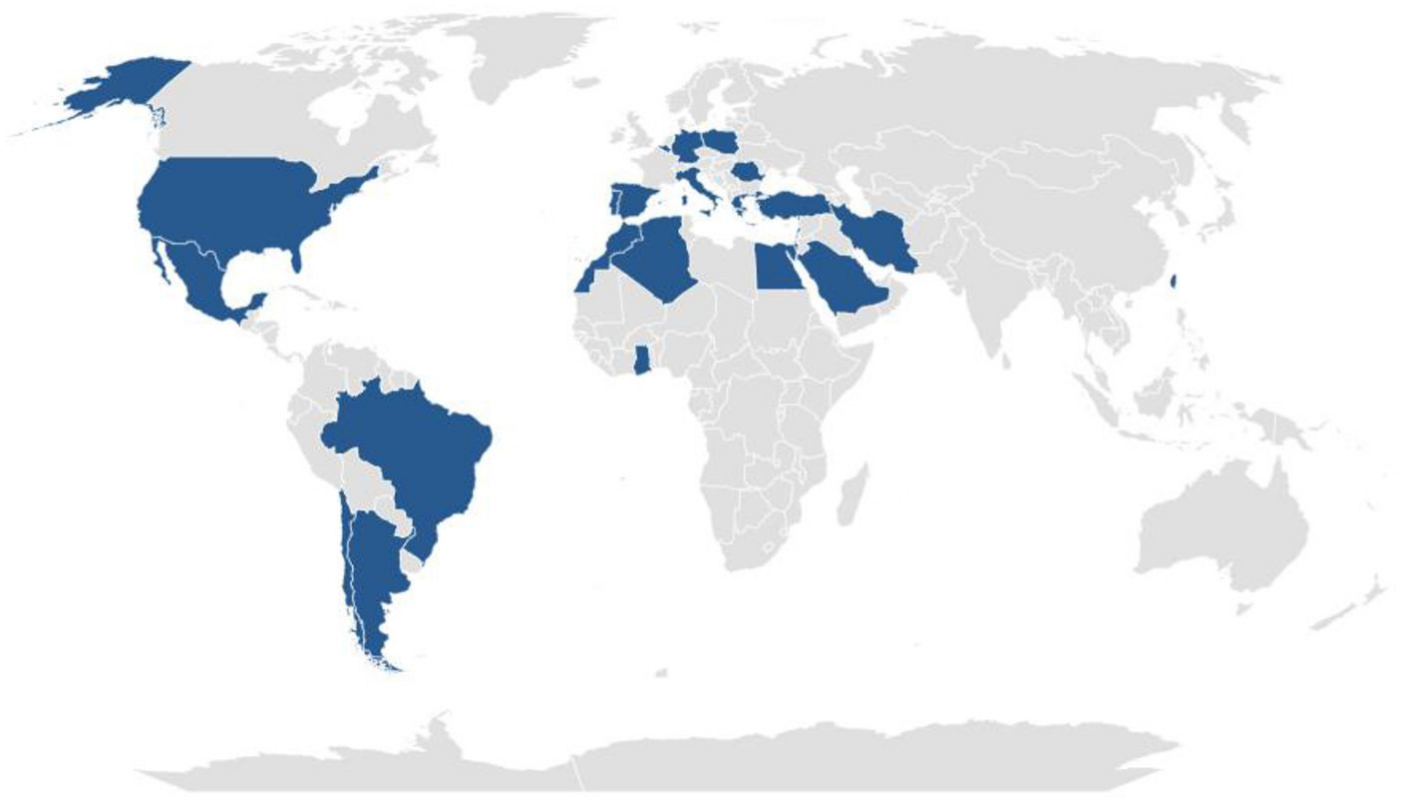
Current geographical coverage of the Autoinflammatory Diseases Alliance (AIDA) Registry of Monogenic Autoinflammatory Diseases. Countries highlighted in violet are those with at least one AIDA partner center (updated to June 20th, 2022).

The AIDA mAIDs registry is a non-population-based registry. The geographic coverage is the catchment area of the clinical centers affiliated to the AIDA Network. The countries with at least one AIDA partner center are shown in Figure 1 (updated to June 20th, 2022). Moreover, the updated geographic coverage of the registry can be found at https://aidanetwork.org/en/clinical-sites.

### Specification of the information to report

The instruments constituting the registry investigate the following sets of variables:

- demographics
- consents
- diagnostic data and family history
- general genetic information:
  - gene mutations

- features of inflammatory attacks:
  - at disease onset
  - between disease onset and diagnosis
  - between diagnosis and the time of enrolment

- clinical diagnostic and classification criteria
- laboratory data
- cardiovascular risk
- past and current treatments:
  - treatment with corticosteroids as main therapy
  - treatment with colchicine
  - treatment with conventional disease-modifying anti-rheumatic drugs
  - treatment with small molecules
  - treatment with biotechnological agents

- fertility and pregnancy
  - disease course and treatment during pregnancies

- follow-up visits: clinical manifestations and treatment
- Death of the patient (to open ONLY in case of patient's death)

The following *common data elements (CDE)* are included in the registry, according to the EPIRARE set of CDE for the European RDR platform (19): patient consent, patient sex, patient date of birth, patient country of birth, diagnosis (standardized according to the OMIM classification), patient country of residence, ID treatment center, other cases in the family (if Yes: degree of kinship), case parents are consanguineous (Yes/No), genetic features of the patient (gene-HGNC Gene Symbol, variant description in HGVS format, variant description in other formats), date of symptom onset, date of final diagnosis, current drug treatment, hospitalizations, patient vital status (and date of death), comorbidity (standardized according to the ICD10 format).

### Registry regulatory status

The ELSI and privacy expertise are provided by the University of Siena. The AIDA project has been firstly approved by the Tuscany Region Ethics Committee - South-East (C.E.A.V.S.E.) area on 24/06/2019 (Ref. N. 14951). The last amendment to the protocol was approved on 02/05/2022. The approval of the protocol should be provided by local Ethics Committee for each of the Centers joining AIDA, whenever required by local regulations. The approval is essential for data collection, but does not affect the participation to the other branches of the AIDA project, such as AIDA Academy or AIDA for patients.

The registry has been developed in accordance with the World Medical Association (WMA) Helsinki declaration 2013 (20) and with ELSI principles and rules, including international and local data protection regulations. The registry conforms to the General Data Protection Regulation (GDPR) (21) ensuring compliance with legal requirements regarding the processing of personal data.

To be eligible for inclusion in the AIDA registry for mAIDs, patients (or their parents/legal guardians) have to provide written opt-in consent. Patients receive from the investigator appropriate information about registry objectives, the type of information collected, how data will be used, the governance and data access rules for third parties and how to withdraw consent at any time.

### Collaborative framework status

The AIDA registry for mAIDs was developed as a specific action of the AIDA Network program (https://aidanetwork.org/en/). Established in 2019, the program aims to move beyond the isolation of reference centers for rare autoinflammatory diseases and autoimmune ocular diseases, facilitating the collection and exchange of clinical data, conduction of multicenter studies and dissemination of scientific knowledge at an international level.

The registry management function and high-level decision making are responsibility of the AIDA Network program governance, chaired by the principal investigator of the AIDA promoter center.

The AIDA registry stakeholders include clinicians, patients, family, and patient organizations (to date, the Italian associations AIFP-Italian Association of Periodic Fevers, ANMAR-National Association of Patients with Rheumatic Diseases, and APMARR-Association of People with Rare Rheumatic Diseases), researchers, the European Reference Network (ERN) RITA.

Inspired by the FAIR guiding principles for scientific data management and stewardship, the AIDA mAIDs registry is included in the European Rare Disease Registry Infrastructure directory (ERDRI.dor, available at https://eu-rd-platform.jrc.ec.europa.eu/erdridor/home) and is committed to promote the interoperability with the other ERN RITA registries and potentially with the other ERNs, within the context of the MeRITA (Metadata registry for the ERN RITA) project (22, 23).

### Informatics infrastructure

The AIDA registry for mAIDs is hosted by a virtual server in the platform of the Laboratory of Bioengineering of the University of Siena, which is located in the Data Center of the Azienda Ospedaliero-Universitaria Senese at the Santa Maria alle Scotte hospital in Siena (Italy).

The registry service is based on REDCap (Research Electronic Data Capture, https://projectredcap.org) a secure web application designed to support data capture for research studies. REDCap provides (i) an intuitive interface for validated data capture; (ii) audit trails for tracking data manipulation and export procedures; (iii) automated export procedures for seamless data downloads to common statistical packages; and (iv) procedures for data integration and interoperability with external sources (24, 25). REDCap requires a typical web infrastructure including one or more secure web servers running a standard software stack (LAMP) that can be deployed “on-premise” (i.e., on the local institution's hardware servers or virtualized servers) or on various cloud-based infrastructures. REDCap can integrate new software modules to extend functionality, such as data query workflow, support direct data capture from patients *via* user-facing surveys and patient pseudonymization. Even though it is not open source, REDCap software is licensed at no-cost for academic, non-profit, and government institutions.

The AIDA Network program developed a standard scheme to build a federated infrastructure of interoperable systems for registry services between partner sites to securely share data and collaborate on research goals. This scheme takes advantage of the portability of project metadata across REDCap installations and on a set of standard operating procedures with rapid turnaround times to: (i) assess, deploy and manage multiple aligned REDCap framework instances and project registries; (ii) efficiently share and deploy standard resources, such as medical data models and ontologies, that already exist or that are newly released by international framework initiatives (such as ERDRI-JRC and ERNs), integrating them into the network of project registries and data collection instruments; (iii) ensure compliance with privacy and security requirements throughout the project partners; and, (iv) guarantee global sharing and FAIR access to data.

### Data management procedure/quality control

The principal investigators of each site are responsible for the validation of data of the corresponding records. They will be also responsible for the accuracy of the information accrued, with the Principal Investigator required to supervise the goodness of overall data. Each Principal Investigator may analyze data gathered in his/her center. Site opening visits are scheduled virtually for each new partner center in order to train the investigators in the correct use of the registry. Further assistance from the AIDA team may be asked by the investigators by email (http://support@aidanetwork.org). The REDCap system allows quality data control at the moment of the data entry by users. In addition, internal data quality audits are periodically performed by the registry staff at the time of sample data extraction or when significant sources of error are identified. The quality control at the time of data extraction is aimed at the control of duplicate records from different investigator sites or logical inconsistencies and range errors on individual data batches sent by each site. When range errors or logical inconsistencies are found, a query is sent to the correspondent principal investigator in order to check the data source. When duplicate records are identified, the record management is dependent on the type of duplication and objective of analysis.

### Security standards and procedures

Data are accessed through secure channels and there are procedures ensuring traceability of their processing, including login authorization procedures and history logs. Confidentiality is ensured on an operational level by pseudonymization and double codification if data are shared, in accordance with the GDPR rules. Full on-site and off-site encrypted data and system back-ups are programmed periodically.

## Results

The AIDA mAIDs registry is running since June 2020.

The registry is composed of 21 instruments and 3,748 fields. By data lock in June 2022, the network counts 118 partner centers and 24 countries in 4 continents and is open to new membership applications. The protocol has been already approved by the local Ethics Committees of 59 partner centers; the remaining EC submissions performed in the last few weeks are in progress or, in a few cases, are not required by local regulations.

At last evaluation (June 20th, 2022), 418 subjects (M:F = 195:212, transsexual *n* = 1, missing *n* = 10) from 35 centers in 11 countries have been enrolled in the registry. Enrolling countries listed in alphabetical order are the following: Algeria, Belgium, Brazil, Egypt, Greece, Iran, Italy, Poland, Romania, Spain, Turkey. Patients' country of birth are Algeria (*n* = 23), Armenia (*n* = 1), Belgium (*n* = 4), Brazil (*n* = 1), China (*n* = 1), Egypt (*n* = 58), Greece (*n* = 6), Iran (*n* = 3), Italy (*n* = 195), Lebanon (*n* = 1), Morocco (*n* = 2), Palestine (*n* = 1), Poland (*n* = 27), Romania (*n* = 4), Spain (*n* = 2), Turkey (*n* = 78), Ukraine (*n* = 1), missing (*n* = 10). Mean age at the time of enrolment is 33.9 ± 16.7 years (range 0–75.9). A positive family history for the same mAID is recorded for 162 subjects (40.9%). Proband's parents are consanguineous in 40 cases (10.2%).

## Discussion

The AIDA mAIDs registry is a powerful infrastructure embedded in the solid collaborative framework of the AIDA program. The registry enables the collection, sharing and valorization of a critical volume of data on mAIDs. Collecting data on autoinflammatory diseases is hampered by the well-known obstacles that rare disease research has to deal with, including the limited number of patients, isolation of research centers, and difficulty in obtaining the correct diagnosis in non-specialized clinical settings. Launched in June 2020, this AIDA registry received favorable attention on the European stage, showing increasing attractiveness, including 118 partner centers from 24 countries in a relatively short time.

According to the results of a survey conducted among the ERN RITA members in 2018, there are twenty-two registries collecting data on monogenic and/or multifactorial autoinflammatory diseases in Europe, with different scopes and geographic coverage: five of them (Eurofever, Infevers, Blau Cohort Study, Pediatric Behçet's Disease Registry, and ImmunAID) are transnational and devoted exclusively to AIDs (in two cases to a single specific disorder); four other registries (ESID, JIR-cohort, Brainworks Study, European Society for Blood and Marrow Transplantation Registry) are transnational and include both autoinflammatory diseases and primary immunodeficiencies and/or autoimmune diseases. The remaining registries collect regional or national data about rare immune diseases, including autoinflammatory diseases, with variable specificity (23). Therefore, it is of the greatest relevance to adopt a standardized scheme and provide a detailed description of the instrument when developing a new registry, in order to ensure adequate quality, efficient interoperability, and long-term sustainability. The AIDA Registry has been developed in such a way to potentially communicate with the existing registries in order to analyze the current clinical and scientific issues from different perspectives. In this regard, the AIDA registry for patients with monogenic autoinflammatory disease is already included within the context of the MeRITA.

In the scene of European registries for mAIDs, the AIDA registry stands out for its disease-specificity, richness in details, flexibility, complex branching logics allowing smart and time-saving data collection, and wide geographic and demographic coverage. With specific regard to the latter, the choice of including subjects of any age naturally follows from the well-established evidence that mAIDs may start from the very first hours of life to late adulthood, and also that adults with pediatric-onset disease may obtain the correct diagnosis with a delay of several decades. The inclusion of adult patients along with children is an asset to this registry, allowing the design of comparative and longitudinal studies, research focused on childhood-to-adult transition and adult-specific issues not yet explored, such as pregnancy, fertility, adult vaccination, comorbidities, long-term disease-related damage, adult-specific outcome measures and PROMs, workability and further socio-economic issues. With this respect, the AIDA actions will be aligned to the research agenda set by the EULAR/ACR points to consider for diagnosis, management and monitoring of the IL-1 mediated AIDs and autoinflammatory type I interferonopathies (26, 27), also in the context of possible future collaborations at the international level.

The registry has been conceived as a flexible and modular tool able to capture the evolving landscape of this field of research. Whensoever new theoretical or practical knowledge is generated, the system enables agile updating of data collection tools. As an example, new modules may be added to include newly identified genes, new treatments that become available or to address to future unmet needs with new research objectives. Moreover, direct queries to the investigators can address specific gaps in the data collection. The AIDA mAIDs registry is inspired by the principles of FAIRness and is committed to adopt the instruments that the EC suggests for the development of registry platforms. Data are standardized by the use of shared libraries such as the ICD-10 and the OMIM classification; the EPIRARE set of CDE for the European RDR platform has been employed when possible (19). The registry has been already registered on the ERDRI directory (https://eu-rd-platform.jrc.ec.europa.eu/erdridor/) and will adopt the new SPIDER tool in the future, with the aim of facilitating the pseudonymization, linkage and transfer of encrypted pseudonymized data among European rare disease registries.

On the other hand, the registry platform supports data capture *via* user-facing surveys and the MyCap application (https://projectmycap.org/), which leverages REDCap, ResearchKit, and ResearchStack tools to capture patient reported outcomes *via* mobile devices. Later, the tool synchronizes the results back to the registry project. The integration of data from the “*AIDA for patients*” action into the AIDA mAIDs registry highlights the huge potentiality of this instrument. Actually, data obtained through the surveys proposed by “*AIDA for patients*” will complement the registry with patient-reported data about quality of life, fatigue, the socio-economic burden of the disease, psychological health, experience of the healthcare pathway and beliefs about medicines. This will allow four-hands studies with the active participation of both patients and their physicians. With this regard, a pilot project co-designed with the patient's association S.I.M.B.A. (*Associazione Italiana Sindrome e Malattia di Behçet*) has been already launched by the “*AIDA for patients*” action for Italian patients with Behçet's disease. A similar experience may be reproduced also in the context of mAIDs and other multifactorial AIDs, with the collaboration of local patient associations (https://aidanetwork.org/en/magazine/aida-for-patients-is-ready-for-launch).

As for the long-term sustainability of the registry, it seems relevant to highlight the lively interest raising from a growing number of international partners both in and outside European borders. Moreover, the AIDA Network program strategic communication and dissemination activities (website, magazine, web events) are equally important to the registry promotion. The program also includes the provision of high-level specialized education in the field of AID, through web-based seminars, face-to-face meetings, and a permanent education archive in collaboration with a renowned international faculty (*AIDA Academy action*). Of note, the program is endorsed by a growing number of patient associations, whose active involvement enhances a multiplier effect, by giving resonance to both AIDA Network program and AIDA mAIDs registry.

Furthermore, the AIDA mAIDs registry has been designed for the implementation of top-down and bottom-up research initiatives. Each Principal Investigator and Site Investigator may provide their study proposals during dedicated meetings. In particular, each Principal Investigator may analyze data collected in their own center for clinical and administrative use. On the contrary, the whole data will be managed by statistics and physicians involved in the network, selected by the Promoter on a case-by-case basis according to their field of expertise. Aggregated CDE are also used for periodic AIDA progress reports and are ready to be shared at the national and European level in the context of the ERNs as publicly indexed metadata and for clinical benchmarking.

In conclusion, we provide a new powerful instrument, the AIDA mAIDs registry, acting both as a research tool for future collaborative real-life studies on mAIDs and as a service to connect all the figures called to participate. These include international researchers, non-specialized clinicians, patients and their representatives, regulatory agencies as well as institutions at the national and supranational level. On this basis, the registry is expected to play a pivotal role in generating new scientific evidence on this group of rare diseases, substantially improving the management of patients and optimizing their impact on the healthcare system.

## Ethics statement

The studies involving human participants were reviewed and approved by Tuscany Region Ethics Committee - South-East (C.E.A.V.S.E.) area on 24/06/2019 (Ref. N. 14951). Written informed consent to participate in this study was provided by the participants' legal guardian/next of kin.

## Author contributions

CG wrote the first draft of the manuscript. AV conceived and designed the study and revised the draft of the manuscript. DR revised the draft of the manuscript. LC conceived and designed the study and accounts for AIDA Registries Coordinator. AB is the bioengineer involved in the technical management of the platform and registries. AT, GR, EA, EW-S, DA-I, GC, LI, MCM, MC, FLT, GL, EV, AP, AS, AI, PPS, AM, MF, BO, DO-B, PP, GE, FS, FCa, MP, JH-R, RP, MA, RNu, AO, ED, PS, PR, FLG, HK, JS, MAH, GM, KJ-R, RS-H, MR, FR, FCi, FI, FD, MF, KL, TG, FF, VSa, DY, VC, MTH, RD, AK, and GK were involved in data recruitment in the Registry dedicated to patients with mAIDs. MT, IA, AL, VM, LD, LB, SGe, FCr, VP, AA-MAM, RNa, MD, CBC, SGr, MM, ID, VSp, HAMG, IV, AR, AF, MAM, BF, GT, and CF were included in the authorship as investigators from the top contributor centers for any of the other seven AIDA Registries. ML was included as leading AIDA expert in the field of mAIDs. All authors contributed to the article and approved the submitted version.

## Conflict of interest

The authors declare that the research was conducted in the absence of any commercial or financial relationships that could be construed as a potential conflict of interest.

## Publisher's note

All claims expressed in this article are solely those of the authors and do not necessarily represent those of their affiliated organizations, or those of the publisher, the editors and the reviewers. Any product that may be evaluated in this article, or claim that may be made by its manufacturer, is not guaranteed or endorsed by the publisher.

## Footnotes

^1^According to Gattorno et al., the genotype is considered confirmatory if 1 pathogenic/likely pathogenic mutation (for CAPS and TRAPS) or 2 pathogenic/likely pathogenic mutations (for FMF and MKD) in the causative genes are detected; the genotype is considered not confirmatory if (1) trans compound heterozygous for 1 pathogenic *MEFV* variant and 1 variant of uncertain significance (VUS), or biallelic VUS, or heterozygous for 1 pathogenic *MEFV* variant is detected, or if (2) 1 VUS in *MVK* or *TNFRSF1A* genes are detected. The pathogenicity of gene variants shall be based on the Infevers classification (10, 13).

^2^According to Shinar et al., the genotype is considered confirmatory when 1 (likely) pathogenic variant in *NOD2, PSTPIP1, TNFAIP3* genes or 1 pathogenic dominant variant in *MEFV* gene or 2 (likely) pathogenic variants in *ADA2* gene are detected; the genotype is considered consistent when 1 novel likely pathogenic variant in *NOD2, PSTPIP1, TNFAIP3* genes or 2 (likely) pathogenic not phased variants in *ADA2* gene or 1 (likely) pathogenic and 1 rare/novel VUS variants in *ADA2* gene are detected; the genotype is considered inconclusive when 1 (likely) pathogenic or 2 rare VUS in *ADA2* or *MVK* are detected (15).
